# Effects of Benzene: Hematological and Hypersensitivity Manifestations in Resident Living in Oil Refinery Areas

**DOI:** 10.3390/toxics10110678

**Published:** 2022-11-09

**Authors:** Raffaele Cordiano, Vincenzo Papa, Nicola Cicero, Giovanna Spatari, Alessandro Allegra, Sebastiano Gangemi

**Affiliations:** 1 Department of Clinical and Experimental Medicine, School and Operative Unit of Allergy and Clinical Immunology, University of Messina, 98125 Messina, Italy; 2 Department of Biomedical and Dental Sciences and Morphofunctional Imaging, University of Messina, 98168 Messina, Italy; 3 Division of Hematology, Department of Human Pathology in Adulthood and Childhood “Gaetano Barresi”, University of Messina, 98125 Messina, Italy

**Keywords:** benzene, VOCs, environmental pollutants, petrochemical plants, hematologic malignancies, petroleum refineries, oil refineries, hypersensitivity, inflammation, asthma

## Abstract

Literature is teeming with publications on industrial pollution. Over the decades, the main industrial pollutants and their effects on human health have been widely framed. Among the various compounds involved, benzene plays a leading role in the onset of specific diseases. Two systems are mainly affected by the adverse health effects of benzene exposure, both acute and chronic: the respiratory and hematopoietic systems. The most suitable population targets for a proper damage assessment on these systems are oil refinery workers and residents near refining plants. Our work fits into this area of interest with the aim of reviewing the most relevant cases published in the literature related to the impairment of the aforementioned systems following benzene exposure. We perform an initial debate between the two clinical branches that see a high epidemiological expression in this slice of the population examined: residents near petroleum refinery areas worldwide. In addition, the discussion expands on highlighting the main immunological implications of benzene exposure, finding a common pathophysiological denominator in inflammation, oxidative stress, and DNA damage, thus helping to set the basis for an increasingly detailed characterization aimed at identifying common molecular patterns between the two clinical fields discussed.

## 1. Introduction

### 1.1. Generalities

Air pollution is an evergreen global public threat. The main contributors to air pollution are volatile organic compounds (VOCs), a group of cyclic hydrocarbons whose main representatives are benzene, toluene, ethylbenzene, and xylene (BTEX). Currently, these compounds are considered ubiquitous environmental pollutants [1,2]. In particular, benzene is a major contributor to air pollutants in the environment, resulting from both natural and human sources. It is mainly used in petroleum and chemical industries to produce fuels, synthetic fibers, plastics, and other commercial products. In addition, it is an intermediate compound during oil processing and is released as a result of tobacco combustion. Cigarette smoke, vehicle exhaust, and industrial emission constitute a significant source of benzene exposure for humans [3]. Benzene is a well-known carcinogenic agent, classified as a group 1 compound by the International Agency for Research on Cancer [4]. Both acute and chronic, benzene exposure also leads to a range of non-carcinogenic effects on various systems impairing hepatic, hematological, and immunological functions, among others [5,6,7,8,9].

### 1.2. Metabolism

**Figure 1:**
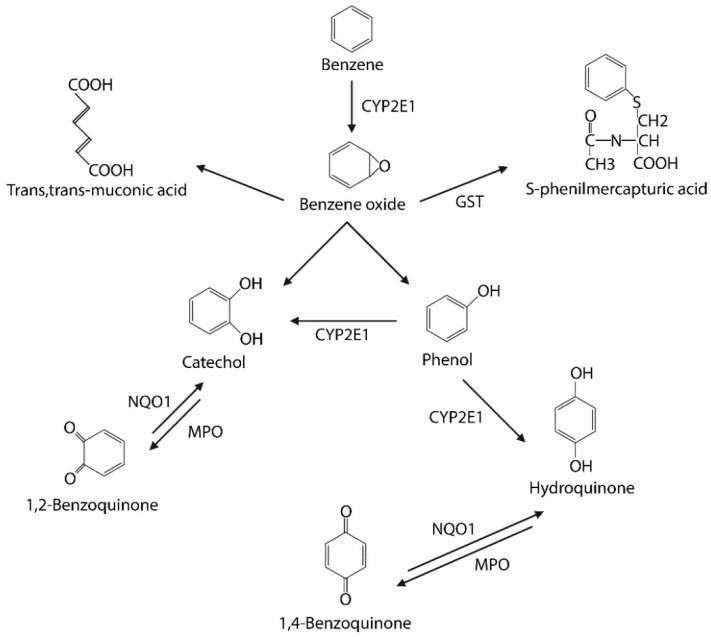
The principal steps of benzene metabolism, its products, and the involved enzymes.

Absorption of benzene occurs mainly through inhalation of polluted air and, to a small extent, through direct contact with skin or mucous membranes and via ingestion of contaminated water or food. The liver is the first organ involved in the metabolism of benzene, which is oxidized by the cytochrome system, mainly by CYP4502E1, into benzene oxide. The latter undergoes various metabolic processes including nonenzymatic rearrangement, hydroxylation, hydration, oxidation, and glutathione conjugation to form various benzene products which the main are phenol, catechol, hydroquinone (HQ), benzoquinone (BQ), and S-phenyl-mercapturic acid (S-PMA). In addition, benzene oxide can be converted into a ring-opening product, trans,trans-muconic acid (t,t-MA). These metabolites undergo phase II metabolic pathways, leading to the excretion of glucuronide and sulphate conjugates, mercapturic acid ring metabolites, and DNA adducts in urine [10]. The second key organ in benzene metabolism is represented by bone marrow (BM). In fact, benzene-derived toxins, including phenol, catechol, HQ and BQ, can reach the BM, which contains several peroxidases, the most prevalent of which is myeloperoxidase, which is responsible for the conversion of these toxins into quinone radicals and the subsequent production of ROS that cause damage to BM hemopoietic stem cells (HSCs) [11]. In addition to the above-mentioned enzymes, others contribute to benzene metabolism, such as GST, GSTM1, GSTT1, GSTP1, and NQO1, leading to further redox imbalance. It is also interesting that the presence of polymorphisms of these enzymatic activities can affect a different metabolism of benzene [12]. The variety of enzymes that co-participate in benzene’s metabolic pathways easily provides an idea of how the presence of polymorphisms can determine a degree of individual susceptibility. The main steps of benzene metabolism are schematically illustrated in Figure 1.

### 1.3. Mechanisms

The effects of benzene on impaired human health have several mechanisms, among which are a state of chronic inflammation, oxidative stress, immune system impairment, DNA damage, and epigenetic changes [13,14,15].

Inflammation is a useful tool for the human body to respond to pathogens and various outsider stimuli [16]. In addition, an adequate inflammatory response aids the healing processes, but chronic inflammation occurs when the trigger is prolonged, or the resolution is inadequate [17]. Inflammation has been widely recognized as a fundamental feature of cancer, and the induction of chronic inflammation is a crucial skill of carcinogens [18,19]. Therefore, the induction of oxidative stress is strongly related to inflammation. Oxidative stress, as previously reported, acts in synergy with other “damaging ways” in benzene-exposed subjects leading to harmful effects. It influences intracellular processes causing alterations such as lipids, proteins, and DNA peroxidation with consequent long-lasting modification [14]. One of the key mechanisms underlying the production of reactive oxygen species (ROS) during benzene metabolism is the activation of pathways catalyzed by cytochrome P450 leading to the formation of benzene reactive metabolites. The latter, once formed, causes oxidative damage by forming covalent bonds with lipids, proteins, and DNA. The resulting adducts interfere with cellular functions by altering the apoptotic phenomena or the expression of genes and proteins. So, the formation of ROS, combined with the direct damage of VOCs on surface receptors, phospholipid bilayers, and intracellular molecular targets, leads to the activation of proinflammatory genes that result in an inflammatory state [20].

As reported earlier, several enzymes contribute to a redox imbalance during benzene metabolism by sustaining a certain level of inflammation that continuously recruits innate and adaptive immune cells producing high levels of proinflammatory cytokines that perpetuate this vicious cycle. The production of ROS and free oxygen radicals during benzene metabolism was associated with the alteration of DNA, damage to DNA-associated protein and chromosomal breaks, and aberration [21,22]. In particular, HQ and BQ are powerful inductors of DNA strand breakage [23]. Alteration in DNA structure and gene expression can be derived not only by modifications in the DNA sequence itself but also by specific processes that fit within the term epigenetic modifications. Methylation, acetylation, phosphorylation, ubiquitylation, and SUMOylation are the main reactions leading to this kind of damage that also influence the DNA reparation system.

### 1.4. Hematological and Hypersensitivity Occupational Manifestations

Hematological and, more recently, hypersensitivity disorders caused by VOCs exposure have been widely studied [24,25,26,27], especially in highly exposed population groups, such as oil refinery workers and residents near refining areas [28,29,30].

Regarding the occupational context, hematotoxicity of benzene has been well-documented in the literature and evidence of work-related hematological consequences is largely conclusive. Many authors have found an increased risk of hematologic malignancies in exposed workers [31,32,33,34]. In addition, altered blood and urinary parameters but also DNA and chromosome damage, have been identified [35,36,37]. The toxic effect on hematopoiesis of benzene exposure affects the proliferation and differentiation of hematopoietic stem and progenitor cells even if the exact mechanism remains unclear [38,39]. The cytokines BM microenvironment and the resulting signaling pathways influence the activity and survival of stem cells, including their propensity to proliferate and differentiate. The impairment of physiological hematopoietic processes is mainly reflected in the reduction in circulating myeloid and lymphoid cells and the consequently compromised function of the immune system [40,41]. Benzene and its metabolites also affect cell function, especially in the host defense capability. Studies have shown that exposure to benzene can induce immunosuppression with a reduction in T-reg cells, NK cells, and B and T lymphocytes and impair some immunoglobulins levels in humans and animals [14,42,43,44,45]. A recent meta-analysis by Zhang et al. [46] showed that the incidence of abnormalities in circulating leukocyte counts was 3.28 times higher in groups occupationally exposed to low levels of benzene than in those not exposed. Decreased function of immunocompetent cells implies decreased immunosurveillance capacity results in increased susceptibility to cancer development [13].

More recent is the evidence in the literature regarding hypersensitivity disorders in oil refinery workers. These are characterized by a relevant inflammatory state [47] and oxidative imbalance that exert their action on the expression of genes directly involved in hyperresponsiveness, airway hyperplasia, and lung remodeling in both atopic and nonatopic subjects. In addition, the same bronchial smooth muscle remodeling resulting from oxidative stress-induced mitochondrial dysfunction leads to an increased susceptibility to chronic obstructive pulmonary disease [48].

In their cross-sectional study, Minov et al. compared the prevalence of respiratory and nasal symptoms, allergic sensitization to common inhalable allergens, and spirometric measurements between petroleum refinery workers and office workers to assess respiratory impairment related to petroleum refinery processes. They found that the prevalence of overall respiratory symptoms was higher in petroleum refinery workers, and a statistically significant difference was found for coughing and wheezing. About objective measurements, a similar prevalence of allergic sensitization to common inhalable allergens was found in both examined groups. At the same time spirometric parameters were lower in the petroleum refinery workers with a statistically significant difference for the maximal expiratory flow at 50% of the forced vital capacity (MEF50) and the maximal expiratory flow at 50% of the forced vital capacity (MEF75) [49].

In a more recent case-control study, Meo et al. found a significant decrease in lung function parameters including Forced Expiratory Volume in first second (FEV1), Peak Expiratory Flow (PEF), Forced Expiratory Ratio (FEV1/FVC%), Forced Expiratory Flow (FEF-25%), and Forced Expiratory Flow (FEF-50%) in petroleum refinery workers compared with the control group. The observed lung function pattern provided evidence of an obstructive lung disease; however, there was no change in the mean value of the Fractional Exhaled Nitric Oxide (FeNO) between the groups [50].

Long-term exposure to benzene, toluene, ethylbenzene, xylene, and styrene (BTEXS) in refinery workers was recently investigated as responsible for the functional decline of the small airways, finding a positive correlation involving in particular benzene among the other pollutants [51].

Since pollutants released by petroleum industries are various and often differ in concentration and composition, other VOCs besides benzene and the concurrence of particular compounds (such as ozone and PM2.5), may be considered in the onset or exacerbations of respiratory and hematological manifestations.

A recent paper by Lawrence et al. [52] revealed that toluene, not benzene, was the main culprit in the onset of asthma in oil spill response and cleanup (OSRC) workers after the Deepwater Horizon disaster in the Gulf of Mexico in 2010. In addition, it has been reported that individuals with allergic asthma are more sensitive to environmental PM2.5 and ozone exposure [53]. A group of Chinese researchers in a recent work [54], using weighted generalized quantile sum (WQS) regression, evaluated the hematological effects of combined benzene, toluene, and xylene (BTX) exposure and the contribution of each pollutant in exposed petrochemical workers. They found a positive correlation between the combined effect of BTX and reduced red blood cell (RBC) and monocyte counts and hemoglobin (Hb) concentration, while the contribution of individual pollutants was positively associated with decreased monocyte and lymphocyte counts and hematocrit (HCT) percentage for benzene, toluene, and xylene, respectively.

Since the categories of subjects exposed to pollutants in refinery areas include residents as well as plant workers, in this study, our purpose is to frame for the first time in a review the comparison between hematological and hypersensitivity manifestations due to benzene exposure, as previously reported often in combination with other pollutants, in resident near oil refineries, with a special focus on the pathophysiological and immunological mechanisms underlying these two pathological spheres of interest.

## 2. Materials and Methods

We carried out a PubMed search including all articles in English published until September 2022. We used the keywords “benzene, VOCs, petrochemical plants, hematotoxicity, hematologic malignancies, residents, petroleum refineries, oil refineries, hypersensitivity” and selected articles concerning residential exposure to benzene in refinery areas, alone or among other pollutants, with no time limit.

## 3. Results and Discussion

The immunoallergic–hematological comparison of the studies found in the literature considers some important parameters, such as the age of the test populations, the distance in kilometers from the petrochemical areas, the exposure time and the outcome of subject exposed to oil refinery pollutants through which the potential acute or chronic effects of the exposures in question can be inferred.

### 3.1. Hematological Side

Eleven studies were conducted evaluating some blood and urinary parameters in residents near petrochemical sites exposed to benzene, among other pollutants.

Six of these referred to a 40-day flaring disaster that occurred in 2010 in a refinery plant in Texas City, USA. This event can be considered an acute residential exposure to VOCs. Indeed, a huge amount of toxic agents, including 7711 kg of benzene, was released into the sky, threatening the health of about 50,000 Texas City residents. Several residential cohorts of exposed people were compared with unexposed control groups living approximately at least 48 km away from the refinery. Both adults and children were included in the studies. The all-aged pilot study [55] included 100 exposed and 100 unexposed subjects. Subsequently, cohort studies were conducted on adults, including on their smoking behavior; indeed, cigarette smoking is the main confounding factor when assessing biological monitoring data of occupational exposure to benzene [56]. In the first work [57], a total of 2213 adults (387 unexposed and 1826 exposed) were recruited, then divided into groups of non-smokers (329 unexposed and 1093 exposed) and smokers (58 unexposed and 733 exposed) subjects and were analyzed in two different studies [58,59]. Children were also enrolled in the cohort studies. Exposed children and adults were compared in a subgroup analysis in the pilot study. In addition, two other papers focused on pediatric subjects considering two cohorts of 312 (155 unexposed and 157 exposed) and 899 (258 unexposed and 641 exposed) children, respectively [60,61].

The pooled results of this acute benzene exposure in all-ages and adult cohorts showed increased white blood cells (WBCs) and platelets (PLTs) count in exposed subjects, regardless of the smoking habit. Moreover, only the exposed non-smokers subjects showed increased Hb and HCT levels compared with the unexposed group. Some liver and renal parameters were also impaired in exposed groups. Regarding pediatric subjects, decreased WBC count and augmented PLT count was found in both cohorts of exposed children compared with controls, while decreased Hb and HCT levels were found only in one cohort study. The subgroup analysis of the pilot study revealed that exposed adults had lower levels of PLTs and higher levels of Hb, HCT, beta2micro-globulin, and urinary phenol compared with exposed children.

Acute exposure to benzene and its hematological effects are not easy to investigate; in fact, few studies exist on this topic. At odds with the above findings, acute exposure to benzene in 15 shipyard workers for 1 to 21 days suggested no significant short-term hematological changes, except for the presence of numerous large granular lymphocytes (LGLs) in 40% of the employees examined [62]. Hematological effects of benzene exposure result in reduced WBC, Hb, and PLT levels, as many studies have noted [40,63,64]. Furthermore, smoking subjects introduce higher burdens of benzene into their bodies [65], so the effects on the hematological compartment should be more pronounced than in non-smokers. The results highlighted by the studies above as being at odds with the well-known effects of benzene exposure are possibly attributable to a certain acute toxicity of benzene or methodological limitations in conducting the studies.

To our knowledge, no studies in the literature support the evidence of increased total WBC and PLT counts and Hb levels in non-occupationally exposed subjects living near petrochemical facilities. However, some studies have revealed altered leukocyte fractions, platelet parameters, and indicators reflecting oxygen transport capacity in subjects exposed to benzene. In fact, a group of Indian researchers found altered leukocyte counts with an abundance of neutrophils, eosinophils, and monocytes with a reduction in T and B lymphocytes in exposed adults compared with controls [66]. A Chinese study showed no significant differences in total WBC, PLT, and Hb values between exposed and unexposed workers. However, an increase was found in some platelet parameters, including platelet distribution width (PDW), mean platelet volume (MPV), and large-platelet cell ratios (L-PCR) in benzene-exposed subjects compared with controls [67]. Finally, Ahmadi et al. found an increase in RBC and Hb values and HCT percentage in gas station workers compared with healthy controls, as often happens when the body requires more oxygen-transport capacity [68].

Some observational studies on the hematologic effects of sub-chronic/chronic residential exposure to benzene were conducted on children. Lee et al. [69] performed a study including 97 children living near a petrochemical site in Korea and 95 children living in a suburban region 16 km away from the polluted area. The exposure time to VOCs, particularly benzene, of the children enrolled was between 4 and 11 years. A complete blood count (CBC) was performed in the study population in three different months. The results showed a decrease in all CBC values in exposed children compared with the unexposed group in the April measurement. In July, a general reduction in CBC values was observed in children residing in the suburban region, resulting in the disappearance of the evidence found in the previous assessment. The authors hypothesized that this latter result might be due to variations in wind direction, traffic activity (the suburban region is more visited in summer) and lower atmospheric pressure in Korea at that time of year. In October, however, decreased Hb and RBC values were found in exposed children. These discordant results suggest that the exposure levels to low levels of benzene, among other VOCs, are different in the study groups over different months. Pelallo-Martinez et al. [70] analyzed the hematological impact of exposure to petrochemical pollutants in children at different industrial sites in Mexico. No differences in hematological parameters were observed between study sites, while a negative correlation was found between urinary t,t-MA levels and WBCs and RBCs counts in children living in the most polluted study area.

Because of their physiology, mature metabolic pathways and clearance systems, children are more susceptible to the effects of toxins exposure compared with adults [71]. These differences regarding pediatric subjects justify the finding of reduced WBC and RBC levels, and Hb concentration, all well-established evidence of benzene exposure [40,63], both in acute and sub-chronic/chronic exposure.

Concerning adult subjects in a chronic setting of benzene exposure, Chen et al. carried out a cross-sectional study among elderly (50–71 years old) residents in Nanjing, located in the most developed region of China [72], where one of the primary activities of the city is the petrochemical industry. Therefore, 240 residents in the polluted area and 181 residents in the control communities were enrolled in the study that analyzed and compared blood benzene, toluene, ethylbenzene, m/p-xylene, and o-xylene (BTEX) levels and other hematological parameters in the two groups. The exposure time ranged from 5 to 71 years. The highest blood levels of BTEX were found in the contaminated group, and the participants’ smoking habits did not influence this result. Besides this, a reduction in neutrophils, PLTs, and RBCs counts, HCT, mean corpuscular hemoglobin concentration (MCHC) levels, and Hb concentration was observed in the contaminated group compared with controls. In contrast, monocyte and basophil counts were significantly higher in exposed subjects than in the unexposed. Finally, there was a significant negative correlation between MCHC and PLT counts and a positive correlation between monocyte counts with blood benzene levels. Another cross-sectional study was conducted by Li et al. in Jilin, China [73]. They selected 499 adults not occupationally exposed to industrial emissions who lived for more than 5 years in the residential area of the Jilin Petrochemical Industrial Park. The authors analyzed blood VOC levels, hematological parameters, and urine indicators in the examined population and, using a binary logistic regression analysis, found that the detection rate of blood levels of benzene was high in the studied group. In addition, a change in urinary WBCs was correlated with blood benzene levels. A Brazilian study [74] evaluated the impact of benzene exposure on the health of residents living near a huge petrochemical complex in Campos Elíseos, Rio de Janeiro State. Urinary S-PMA and genetic polymorphism for CYP2E1 1293G>C and NQO1 609C>T were determined in 190 adults living within 1 km of the refinery for more than 3 months. Twenty-one subjects had a quantifiable presence of urinary S-PMA, associated with specific hematological abnormalities, particularly in neutrophil count and mean corpuscular volume (MCV) levels. CYP2E1 and NQO1 are crucial enzymes in benzene metabolism. The first one, a phase I enzyme, exerts its functions in several organs, mainly in the liver, where it converts benzene and other benzene-derived metabolites into other toxic compounds. So, the greater the activity of CYP2E1, the greater the production of benzene-derived toxins. NQO1, a phase II enzyme operating mainly in the BM, reduced BQs to less toxic metabolites. For this reason, the higher the activity of these enzymes, the lower the toxicity exerted by benzene metabolites. The presence of a polymorphism in the promoter region at 1293 of CYP2E1 is associated with increased enzyme activity, while a homozygous genotype for a C→T base substitution at position 609 of the gene encoding for NQO1 can show the absence of enzyme activity [75,76,77,78,79]. The results of the above study showed a statistical correlation between the presence of the variant allele in the NQO1 genotype and the hematological abnormalities observed in exposed adults compared with controls.

Several studies have reported a reduction in some blood parameters in adults chronically exposed to benzene, including WBC, RBC, and PLT levels and total neutrophils count [40,80,81,82]. These values consider the toxicity exerted by benzene on BM myeloid and lymphoid stem cells. In addition, urinary metabolites such as t,t-MA and S-PMA have been evaluated as suitable biomarkers of benzene exposure. In particular S-PMA was reported to be a more specific, sensitive, and accurate marker than t,t-MA [56]. Therefore, the results of the mentioned studies on chronic benzene exposure in residents near petrochemical areas are consistent with the described evidence above.

Numerous epidemiologic studies have evaluated the incidence, prevalence, mortality, and risk of hematologic malignancies in residents near petrochemical plants. An ecological study by Barregard et al. showed an increased incidence of leukemia cases (33 cases vs. 22 expected cases) but not lymphoma cases, between 1975 and 2004, in residents near a petrochemical plant in Lysekil, Sweden. Specifically, 11 cases were diagnosed as chronic lymphocytic leukemia (CLL), 7 cases as acute non-lymphatic leukemia (ANLL), a synonym for Acute Myeloid Leukemia (AML), and 15 cases of “other” leukemia, including acute lymphoblastic leukemia (ALL), chronic myeloid leukemia (CML), myelofibrosis (MF), and polycythemia vera (PV). The annual average benzene concentration emitted by the refinery was estimated to be 2 μg/m^3^ [83]. In contrast, another Swedish study with almost the same observation period (1975–2004) found that the number of leukemia and lymphoma cases was similar to that expected for residents near the Stenungsund petrochemical plant. In this case, benzene emissions remained fairly stable over the years, with an estimated concentration of 0.3 μg/m^3^ [84]. Evaluation of cancer incidence among residents near five refineries from 1973 to 2006 in Utah, USA, showed increased risk only for non-Hodgkin’s lymphoma (NHL), while no evidence of increased risk of leukemia, multiple myeloma (MM) or Hodgkin’s lymphoma (HL) were found. The study did not specify the subtype of hematological malignancies that occurred nor the concentration of VOCs, particularly benzene, emitted by refineries [85]. Hurtig et al., in their ecological study, found an increased risk of childhood leukemia in exposed subjects [86]. Weng et al. found the same evidence among children and young adults (<=19 years old) in Taiwan, showing an increased risk of childhood leukemia incidence [87]. Young people in Taiwan were also studied by Yu et al. They divided the survey population into two groups (0–19 and 20–29) to assess the risk of childhood and adult leukemia. Unlike the previous Taiwanese study, the results showed that there was no association for the younger age group, while an increased risk of leukemia was found for the older age group [88]. Two different studies have been conducted in South Wales. Lyons et al. found no significant excess of leukemia and lymphoma cases between 1974 and 1991 in children and young people residing within a 1.5–3 km radius of a refinery where benzene concentration in the air ranged from 4 to 16 ppb. In contrast, Sans et al. reported an increased incidence of all cancers, including some hematological malignancies, in the study area (7.5 km radius from another refinery) where the average benzene concentration was about 1–5 ppb [89,90]. An Italian study assessed mortality from hematologic malignancies and found an increased risk of death from leukemia or NHL in women and the retired–homemakers–unemployed category living closer to refineries. Moreover, these risks were positively associated with proximity to factories [91]. Another group of researchers evaluated the incidence of cancer diseases in residents of a town in northeastern Italy about 3 km away from a refinery. They found an increased incidence of MM in women and leukemic lymphoid neoplasms in women and men [92].

Dahlgren et al. found an increased prevalence of Hodgkin’s disease in residents 1.6 km away from a refinery in Sugar Creek, Missouri, USA [93]. Finally, Ramis et al. indicated a possible increased risk of mortality from NHL in residents near 10 refineries in Spain without showing a gradient in relation to increasing distance from the plants [94]. 

The main features of the considered hematological studies are shown in Table 1.

### 3.2. Hypersensitivity Side

Interest in the study of hypersensitivity diseases in petroleum area residents arose and grew in response to the positive findings on the occurrence of these diseases in oil refinery workers [49,50]. However, in the international literature, also considering the narrow target population on which we are focused, there are currently only works available investigating respiratory hyperresponsiveness, with a lack of papers focusing on skin hypersensitivity, which finds its main clinical expression in atopic dermatitis.

Regarding residential respiratory hypersensitivity manifestations due to refineries contaminants, eight research groups have dedicated themselves to the issue, focusing on benzene among the various other pollutants examined. The number of publications included in this part of the review refers to the decade 2009–2019. No other relevant publications have been found in previous years. This helps us to understand that the interest in this topic is relatively young. Equally young is the population examined.

In fact, all the studies were conducted on children and adolescents, supporting the most consolidated idea of allergy as a disease of the youth, even if many studies, including recent ones [95,96], aim to dispel this erroneous belief.

All the revised studies were conducted in highly representative areas of industrial plants and, in particular, of oil refineries in the European, Asiatic, and American continents.

Concerning the observation time of the population studied, short- [97], medium- [98,99,100,101] and long-term [102] monitoring is available.

Wichmann et al. were the first to take an interest in the subject [98]. Their strategic choice was to conduct their case-control study in 2005–2006 in La Plata, Argentina, due to its location next to the most important oil refinery in the country, constituted by the association of 6 oil plants located about 10 km from the urban core. The target population of the study was 1212 children (6–12 years old) residing in four different regions with different pollution rates: industrial area close to oil plants, urban area exposed to heavy traffic, “semi-rural” and “residential” regions, probably not polluted. The study aimed to evaluate the effects of exposure to airborne particulates and VOCs on the respiratory health of the recruited subjects with an approach aimed at assessing, rather than short-term acute effects, the long-term effects of cumulative exposure to pollutants. Health and demographic information were obtained from parents by using a questionnaire modified from the International Study on Asthma and Allergies in Children. Standard spirometry was used as a pivotal tool to assess the lung function of 181 randomly selected children. Air sampling and chemical analysis were conducted in 4-week periods during the winter months of 2005 and 2006. Regarding benzene, its average concentration detected in the industrial area (19.3 μg/m^3^) was significantly higher than in the control regions. This can largely explain the findings of this cross-sectional study. In addition, a higher prevalence of asthma was observed, as well as asthma exacerbations and respiratory symptoms in children residing in industrial areas and, therefore, more exposed to petrochemical pollutants. These findings were also accompanied by a decline in lung function with spirometric evidence of significant deficits in FVC, FEV1, FEV1/FVC ratio, and FEF25-75. Furthermore, the length of residence in an industrial area was considered an important factor influencing respiratory function decline.

In the same year, de Moraes et al. [99] conducted a smaller cross-sectional study designed to assess the possible association between exposure to industrial pollutants and the appearance of wheezing in 209 children and adolescents aged 0–14. The target population, since 2006, lived for at least one year within a radius of 5 km from the petrochemical complex of Guamaré in Rio Grande do Norte, Brazil. For this purpose, information was collected regarding the respiratory health of the recruited subjects: diagnosis of asthma, wheezing and its frequency in the last 12 months, alteration of sleep quality, and nocturnal dry cough with non-infectious etiology. These data were supplemented with other relevant socio-demographic information and domestic tobacco smoke exposure. The ISAAC questionnaire was used for the detection of asthma in schoolchildren [103]. The level of benzene (32.4 ± 9.9 µg/m^3^), among other pollutants, was measured by a monitoring station set up approximately 4 km from the petrochemical complex and downwind of it. Although the environmental levels of monitored pollutants were consistently within acceptable limits for the duration of the study, respiratory symptoms were more frequent in children and adolescents living in exposed communities. In addition to place of residence, male gender and age under 7 years were also risk factors for wheezing among inhabitants of the Guamaré Petrochemical Complex area.

Along the lines of the previous study, an Italian cross-sectional study was conducted by Rusconi et al. in Sarroch (Sardinia, Italy), a small municipality close to one of the biggest high-complexity refineries in the Mediterranean and to the largest European liquid fuel gasification plant [100]. Their case-control survey aimed to evaluate air pollution’s role on lung function, oxidative stress, and inflammation by comparing children living near an oil refinery (municipality of Sarroch) with those living in an unpolluted control area (municipality of Burcei). A panel of 54 children and adolescents consented to participate in a longitudinal survey (January–June 2007) to study the lung function and FeNO test concerning air pollution. Data were collected using the ISAAC questionnaire (completed by 275 children and adolescents aged 6 to 14 attending primary and middle schools in the municipality of Sarroch) to study wheezing symptoms. Moreover, the Studi Italiani sui Disturbi Respiratori nell’Infanzia e l’Ambiente (SIDRIA) questionnaire [104] was used to collect data regarding individual and family characteristics, respiratory symptoms other than asthma, and children’s exposure to known or suspected risk factors. The tools used were spirometry (with a focus on FEV1 and FEF25–75), FeNO test, and the analysis of Malondialdehyde–deoxyguanosine (MDA–dG) adducts from the nasal mucosa. Weekly average concentrations of benzene and other pollutants were assessed by passive dosimeters located on a regular grid of 21 locations in the municipality of Sarroch and at the primary and middle schools in Burcei. Regarding benzene concentration, the median rate was 3.7 µg/m^3^ in Sarroch and 1.4 µg/m^3^ in Burcei. A decrease in lung function and increased markers of bronchial inflammation and oxidative DNA damage were found in children living near an oil refinery compared with those living in an unpolluted control area. Moreover, MDA–dG adducts values tended to be higher among the children living in Sarroch than those living in the rural area of Burcei. Asthma symptoms in the last 12 months also differed in the two areas.

Smargiassi et al. conducted a panel study [97] of children with asthma living in proximity to an industrial complex housing two refineries in Montreal, Quebec, in order to assess the associations between daily exposure to benzene and other air pollutants and changes in pulmonary function and selected indicators of cardiovascular health in the target population. Seventy-two asthmatic children aged 7–12 years were enrolled in 2009–2010 for 10 consecutive days. They carried a small backpack for personal monitoring of benzene and other pollutants and underwent daily spirometry and cardiovascular testing (blood pressure, pulse rate, and oxygen saturation). Mean personal exposure to benzene was 2.80 µg/m^3^. In asthmatic children living in an industrial area, there was no evidence of associations between pulmonary function, oxygen saturation, pulse rate, and blood pressure and personal daily concentrations of exposure to ambient air pollutants, including benzene. So, it was reasonable to conclude that these effects may be difficult to detect over 10 consecutive days.

Tanyanont et al. conducted a population-based cross-sectional study in Map Ta Phut Industrial Estate (MTPIE), in Rayong Province, the largest petrochemical industrial complex in Thailand, housing 60 petrochemical plants [105]. Obtaining data from a standardized questionnaire investigated whether residence proximity to MTPIE was associated with chronic respiratory or irritant health problems. Of the individual compounds, the average concentration detected of benzene was 2.80 µg/m^3^. The results showed that those who lived longer than 5 years near the MTPIE had an increased risk of dyspnea and wheezing. Furthermore, older adults (>40 years) were more affected by chronic respiratory problems, lower respiratory tract symptoms, and eye irritation.

In their comparative study, Rovira et al. [106] aimed to estimate the prevalence and severity of asthma as well as respiratory and allergic symptoms and to evaluate the lung function of children (total number of 1544 with the target age ranges of 6–7 and 13–14) living near the petrochemical sites of Tarragona (where is located the largest chemical site in southern Europe and the Mediterranean region) and children living in areas with urban pollution compared with children living in relatively unpolluted areas of the county. Health information was obtained using the ISAAC questionnaire; demographic and environmental data were also collected. Lung function was measured using a portable spirometer. According to the local air quality monitoring network, benzene’s average concentration range for 2010 was 0.9–3.7 mg/m^3^. Unexpectedly, a higher prevalence of asthma and allergic symptoms was not found in children and adolescents living near a petrochemical site compared with those living in the control area. Moreover, unlike the two previously cited studies performing spirometry [98,100], they did not find a lower lung function, while an increase in hospital admissions for respiratory symptoms and nocturnal cough was noted in the enrolled children, which can be related to the exposure to industry-derived petrochemicals.

The most recent studies on the subject are two European works, both conducted in Eastern Estonia, which is among the areas in Europe with the highest density of oil shale mines and industrial facilities mainly focused on electricity generation from oil shale and shale oil extraction [101,102].

In their case-control study Orru et al. [102] aimed to identify the air quality situation in Ida-Viru County (the region with the largest air-polluting industry in Estonia) and people’s annoyance regarding air pollution to study the relationship between industrial contaminants and the residents’ health. A total of 2127 people responded to the study of the health impact of oil shale sector (SOHOS) questionnaire. All participants received a postal questionnaire about their socio-economic situation, general health, respiratory symptoms, chronic diseases, indoor and outdoor environment, working history, and health behavior. The study analyzed Ida-Viru County monitoring data from 2001 to 2013, with the subsequent characterization of pollutant levels to quantify population exposure throughout the region. Annual average benzene in the ambient air of Ida-Viru County was significantly higher. This can partially explain why, compared with the control groups from non-industrial areas of Tartu or Lääne-Viru County, residents of Ida-Viru County more frequently reported wheezing, chest tightness, shortness of breath and a long-term cough, which are characteristics of acute asthma exacerbation, and cardiovascular diseases such as hypertension, heart diseases, myocardial infarction, stroke, and diabetes. People living in regions with higher levels of benzene and other industrial pollutants had higher odds of experiencing chest tightness, shortness of breath, or asthma exacerbations and a long-term cough or myocardial infarction during the previous years.

In the wake of this latest study, Idavain et al. [101] examined the associations between respiratory symptoms, asthma, increased levels of FeNO and ambient levels of industrial pollutants (such as benzene, phenol, formaldehyde, and non-methane hydrocarbons) for schoolchildren living near oil shale industries in Ida-Viru County, Estonia. The study population consisted of participants in two different studies: Schools Indoor Pollution and Health: Observatory Network in Europe (SINPHONIE) and SOHOS. They randomly selected 1326 schoolchildren from twenty-five schools, aged between 8 and 12 years and living in northeastern Estonia (in Ida-Viru and LääneViru Counties) or southeastern Estonia (Tartu County). Questionnaires on health and socio-demographics were distributed to subjects. Survey respondents were then invited to a clinical examination, during which the content of FeNO was determined as a marker of eosinophilic airway inflammation. A total of 1208 subjects completed the questionnaires, and 1098 attended the clinical examinations. It was seen that children exposed to higher levels of industry-specific air pollutants, such as benzene, had significantly higher odds ratios (ORs) for most of the tested symptoms: rhinitis, asthma recrudescences, dry cough without phlegm during the night over the last 12 months during the year; as well as increased prevalence of high values of FeNO (≥30 ppb). So, the prevalence of symptoms commonly associated with asthma, and asthmatic symptoms, were higher among 8–12-year-old children in industrially exposed Ida-Viru County, compared with the non-industrially polluted Tartu region. Table 2 resumed the main characteristics of the considered studies.

### 3.3. Immunological Aspects

There are some common and mutually influenced pathophysiological mechanisms induced by VOCs, among which benzene is certainly a fundamental trigger, responsible for the onset of hypersensitivity and hematological disorders such as inflammation, oxidative stress, DNA damage, and epigenetic changes.

The main parallelism from the point of view of molecular mechanisms between the two branches under consideration mainly involves the cellular signaling pathways-related cytokine networks and how they are pathologically affected by benzene exposure.

On the hematological side, benzene exposure has been associated with an increase in pro-inflammatory cytokines (IL-6, IL-8, TNF-α, IFN-γ, and IFNB1) and chemokines (CXCL16) [13]. IL-4 and IL-5, two main Th2 cytokines, were augmented in PBMC cells exposed to benzene metabolites catechol, HQ, and p-BQ. Moreover, in the same study, the authors found suppression of the anti-inflammatory cytokine IL-10; these findings reinforce the idea of a relevant inflammatory state during exposure to benzene [107]. Conversely, in a more recent study, IL-10 was augmented in benzene-exposed workers in association with an increased level of IL-4, IL-15, TNF-α, and VEGF and a decreased level of IL-9 compared with non-exposed subjects [108]. As previously reported, HSCs residing in the BM are strongly affected by the toxic effect of benzene metabolites. Indeed, activating various signaling pathways in the BM microenvironment alters the differentiation and proliferation of these stem cells, resulting in a harmful effect on hematopoiesis [109]. Many pathways were studied to explain this toxic effect on the hematopoietic system.

The nuclear factor-kB (NF-kB) pathway, which promotes cell proliferation and survival and plays a critical role in carcinogens of multiple tumors, was involved in the hydroquinone-induced double-strand break (DSB) homologous recombination (HR) repair mechanism in human osteosarcoma cell line (U2OS). The authors observed increased expression of mRNAs related to NF-kB target genes (BIRC3, CCL2, CCL20, and CD83) and proteins (phosphorylated-p50, -p52, and -p65) following hydroquinone-induced repair [110].

Sun et al. [111] found an altered activity of mitogen-activated protein kinase (MAPK) pathway with an upregulation of 5 genes and 32 downregulated genes related to this pathway in exposed mice HSCs, suggesting that alteration of the MAPK pathway may contribute to the benzene-related toxic effects. They also valued the genetic setting in peripheral blood HSCs (PBMCs) of the same animal model exposed to benzene and observed a significant change in the expression of 3 genes (Cd7, Cd9, and Cd22) that are related to the differentiation of lymphoid stem cells into T and B lymphocytes. Additionally, the expression of 10 genes associated with the differentiation of myeloid stem cells including, 1 gene for monocytes (Cd24), 3 genes related to RBCs (GypA, Daf, and Cd59), and 6 other genes associated with platelets (Itgb3, Cd9, Cd14, Itga2B, Gp5, and Itga1) was altered in the investigated cells. Moreover, in an animal cell model exposed to BQ, the activation of MAPK pathway enhanced the NF-kB-related signaling [112].

The nuclear factor erythroid 2 (Nrf2)-related pathway, involved in gene expression of antioxidant enzymes and maintenance of cellular redox balance, was also studied. Nrf2-/- (Nrf2-KO) mice exposed to low doses of benzene showed relief in peripheral blood cell count decrease despite an increase in oxidative stress and DNA damage, indicating the involvement of Nrf2 in the regulation of benzene-induced hematotoxicity through oxidative stress-mediated pathway [113]. In addition, a more recent animal study shows a decrease in cytoplasmic Nrf2 and an increase in nuclear Nrf2 following subcutaneous injection of benzene in mice, suggesting activation of the antioxidant role of the Nrf2 pathway in the hematotoxic effect elicited by benzene [114].

Using a murine model, Malovichko et al. examined endothelial injury-related particles and inflammation markers due to benzene exposure. They found a significant increase in circulating and activated endothelial and platelet microparticles and platelet-leukocyte aggregate, an expression of altered endothelial cell cycle and platelet activation. In particular, among many benzene metabolites, t,t-MA strongly increased the activation of pan apoptotic markers (caspase-3, -7 and -9). Moreover, an augmented activation of the NF-kB pathway, inflammatory response, leukocyte cell–cell adhesion and apoptosis were noted in the liver of the exposed animals [115].

Lu et al. [109] reviewed some signalling pathways associated with hematopoiesis and leukemia relating their alterations to the well-known carcinogenic effect of benzene. Specifically, the Hedgehog and Notch/Delta pathways (involved in proliferation and maintenance of potency of stem cell), Wingless/Integrated pathway (associated with some tumors such as melanoma, colorectal and breast cancer, and leukemia), and NF-kB pathway (regulating genes involved in cell proliferation and apoptosis besides being intrinsically involved in the pro-inflammatory response).

**Figure 2:**
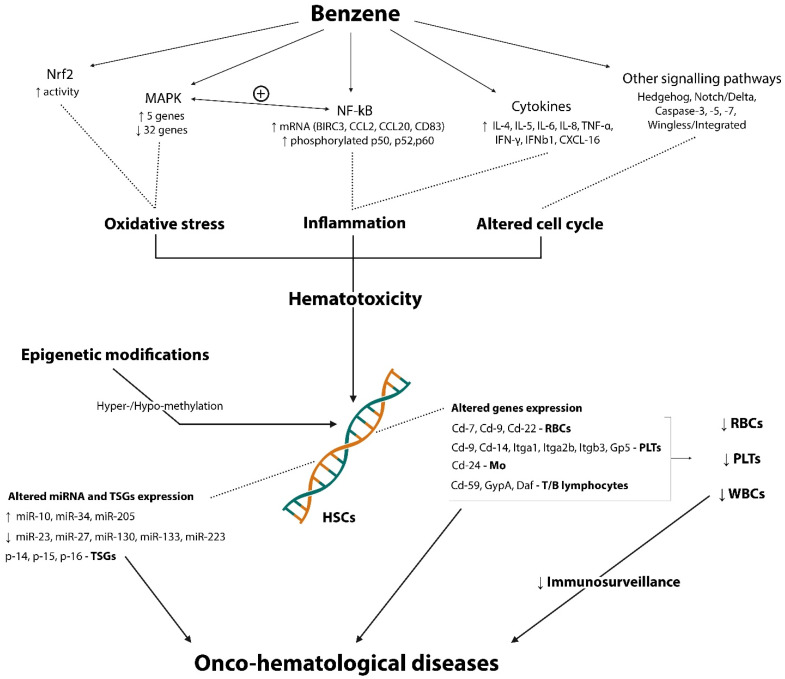
Schematic representation of involved pathways, mechanisms and effects on HSCs exerted by benzene in hematological compartment.

The epigenetic effects of benzene exposure have been studied mainly concerning hematologic diseases. Recently, Spatari et al. [116] conducted a review collecting the main evidence correlating epigenetic changes with the occurrence of onco-hematological diseases during benzene exposure. DNA and histone hyper- and hypo-methylation changes that alter chromatin structure and gene expression, particularly in tumor suppression genes (TSGs) p14, p15, and p16, which are essential in regulating cell growth, and miRNA expression such as miR-10, miR-23, miR-27 miR-34, miR-130, miR-133, miR-205, and miR-223 were the main epigenetic mechanism arisen by the authors. This latter miRNA was positively correlated with indoor benzene exposition with a reduction in T-reg cells in pregnant women’s blood [45]. Figure 2 summarizes the main treated immunological aspects of benzene exposure related to the hematological field.

Regarding atopy, the primary effector cells (mast cells and basophils) involved in the onset of hypersensitivity diseases such as rhinitis and bronchial asthma are directly or indirectly influenced by exposure to benzene and its metabolites [117]. In fact, in vivo exposure to low levels of HQ is related to increased production of TNF by tracheal epithelial cells with the direct consequence of increased responsiveness and, therefore, contraction of smooth muscle cells in response to a cholinergic agent. Moreover, mast cell degranulation induced by TNF secretion contributes to maintaining HQ-induced tracheal hyperresponsiveness, thus influencing the onset of airway diseases [118].

There is evidence [119] that exposure to benzene can reduce counts of IFN-producing type 1 cells (particularly in the CD8+ subpopulation of T cells). On the other hand, it increases counts of IL-4-producing type 2 cells (mainly in CD4+ T cells).

Since the reduced secretion capacity of type 1 cytokines (especially IFN-γ) increases the risk of developing allergic manifestations in childhood [120], this explains why children and adolescents residing near oil refineries are the most at risk, and for this reason, most studied.

Regarding the influence on gene expression, benzene-extracted components can also determine a significant upregulation of IL-8 mRNA levels in human peripheral airway epithelial cells [119]. Th2-cytokines, particularly IL-4 and IL-5, contribute to the pathogenesis of asthma [47]. Furthermore, some articles have correlated the serum level of IL-8 in patients with severe atopic asthma with disease activity [121,122]. We can therefore hypothesize a key role of benzene in stimulating the production of these specific cytokines and related networks responsible for the state of hypersensitivity that characterizes asthma.

Chronic exposure to benzene was correlated with the occurrence of excessive DNA damage in airway inflammatory and epithelial cells in biomass smoke-exposed [123]. Even considering that this damage is probably mediated by oxidative stress, we can reasonably consider the appearance of the same type of damage and similar pathogenesis in subjects exposed to benzene residing in refinery areas.

About lung damage, an in vitro study [124] of human lung cells exposed to benzene showed an increase in mRNA expression of matrix metalloproteinases MMP-2 and MMP-3 associated with a downregulation of tissue inhibitor of metalloproteinases TIMP-1 and TIMP-2 suggesting a dose-dependent increase in pro-destructive activity as early dysfunction occurring in the airways of exposed subjects.

**Figure 3:**
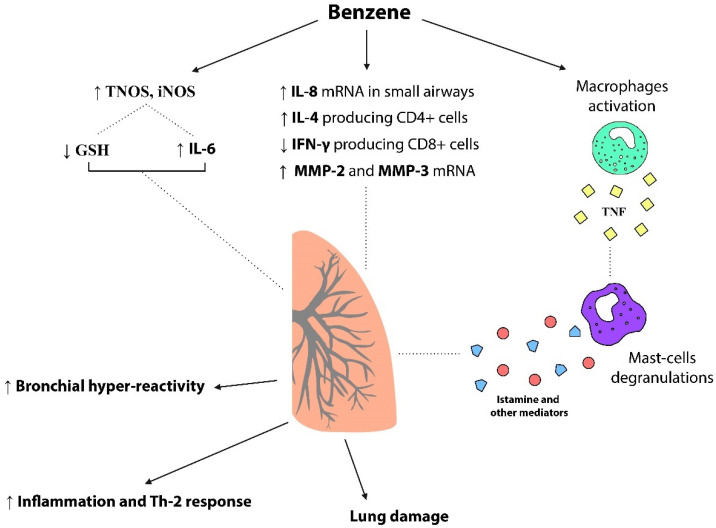
Respiratory-related hypersensitivity effects and immunological actors in benzene exposure.

Regarding redox imbalance, [125] the effect of short-term exposure to a high dose of VOCs was studied in mice and a significant increase was found in total nitric oxide synthase (TNOS) and inducible form of NOS (iNOS) activity, which in turn resulted in a consistent pro-inflammatory stimulus through GSH reduction and increase in IL-6, involved in airway inflammation and bronchoconstriction. Furthermore, a study displayed that the AOPPs serum levels in oil refinery employees were statistically significantly higher than in controls [126]. These findings were associated with a significant reduction in IFN-γ. As further confirmation, the eosinophil count, the main cells involved in the induction of oxidative stress in the airways, was increased. On the other hand, IL-4 levels were not significantly increased. Immunological pathways regarding hypersensitivity manifestations are illustrated in Figure 3.

### 3.4. Hematological Highlights

- Involvement of many pathways (NF-kB, Nrf2, MAPK, Hedgehog, Notch/Delta, Wingless/Integrated, Caspase-3, -7, -9);
- Augmented pro-inflammatory cytokines (IL-6, IL-8, TNF- α, IFN-γ, and IFNB1) and chemokines (CXCL16);
- Augmented Th2 cytokines (IL-4, IL-5) and controversial role of IL-10;
- Changes in the expression of genes related to the differentiation of T- and B- lymphocytes (Cd7, Cd9, and Cd22) and with the differentiation of myeloid stem cells (Cd24-monocytes; GypA, Daf, and Cd59-RBCs; Itgb3, Cd9, Cd14, Itga2B, Gp5, and Itga1-PLTs);
- Epigenetic modifications impairing TSGs (p14, p15, p16) and affecting miRNA expression (miR-10, miR-23, miR-27, miR-34, miR-130, miR-133, miR-205, miR-233);
- Reduction in WBCs, PLTs and RBCs count in exposed subjects;
- Discordant results about Hb concentration and HCT percentage in acute exposure and reduction in chronic exposure.

### 3.5. Hypersensitivity Highlights

- Increased production of TNF elicited by HQ and consequent mast-cell degranulation involved in hyperresponsiveness;
- Reduced counts of IFN-γ producing type 1 CD8+ subpopulation of T cells and increased counts of IL-4 producing type 2 CD4+ T cells benzene-induced;
- Benzene-induced upregulation of IL-8 mRNA levels in small airways epithelial cells;
- DNA damage in airway inflammatory and epithelial cells related to chronic benzene exposure;
- Benzene-related lung damage due to upregulation of matrix metalloproteinases (MMP-2 and MMP-3) and downregulation of their inhibitors (TIMP-1 and TIMP-2);
- Association of GSH reduction and increase in IL-6 as effects of short-term exposure to a high dose of VOCs determine airway inflammation and bronchoconstriction.

## 4. Conclusions, Recommendations and Future Goals

Benzene exposure has harmful health effects, especially in highly exposed population groups, including workers in certain industrial sectors. Similarly, the scientific world’s attention should not be limited to them but should also extend its interest to non-workers residing in just adjacent industrial areas. The mechanisms through which benzene harms human health are not fully understood. The continuing research is tasked to discover these processes to protect the exposed population, workers, and non-workers alike.

Our review revealed a key role of benzene in determining harmful effects on residents of refinery areas related to hematological and hypersensitivity fields, pathological spheres only seemingly disconnected from each other. In particular, laboratory examinations (RBCs and WBCs count, some PLTs parameters, Hb values, and urinary metabolites of benzene), epidemiological studies on the occurrence of onco-hematological diseases, and the evaluation of respiratory function indices of benzene-exposed individuals must be provided the utmost attention by the scientific community and the relevant authorities. Since in many of the considered studies, benzene was not the only VOC released into the area by oil facilities, it is not justifiable to attribute the harmful effects described in this review to this compound alone. However, it certainly has a central role, as it is also the compound that has been most studied over time, especially regarding onco-hematological occurrences.

It is essential to standardize the methods of monitoring the environmental concentration of pollutants and the strategies by which studies, especially epidemiological studies, are conducted to obtain data that can be easily interpreted and compared. The main goal of such a strategic choice would be to achieve, with a single environmental intervention, optimal primary prevention of both onco-hematological diseases and hypersensitivity diseases, with a consequent reduction in the important social costs and health care expenditures associated with the management of these chronic conditions.

However, it must be said that to date, based on what has been found in the international literature, also considering the level of detail of the studies examined on the subject, which do not allow more specific comparisons, it is not yet possible to elaborate a meticulous treatment regarding specific molecular mechanisms that clearly correlate the onset of each of the two pathological spheres treated, except for the great common pathophysiological denominator represented by inflammation, oxidative stress, DNA damage, and epigenetic changes. For this reason, the highlights reported here are proposed as an ambitious forerunner for a more detailed characterization of the immunological links between these two clinical branches.

## Figures and Tables

**Table 1:** Main hematological studies and their characteristics found through our research.

Author	Date	Refineries Site	Cohort	Age	Distance from Refineries	Exposure Time	Observational Time	Outcome in Exposed Group
*Data studies*								
D’andrea et al. [55]	2013	Texas City, USA	200 (100 exposed)	All	<48 km	40 days	2010–2012	↑ WBCs, PLTs, ALP and AST; ↓BUN and creatinine. Adults vs. Children (exposed): ↑ Hb, HCT, ALT, creatinine, beta2-microglobulin and urinary phenol; ↓ PLTs and ALP
D’andrea et al. [57]	April-16	Texas City, USA	2213 (1826 exposed)	Adults	<48 km	40 days	2010–2012	↑ WBCs, PLTs, creatinine, ALT, AST and ALP
D’andrea et al. [58]	December-14	Texas City, USA	1422 non-smokers (1093 exposed)	Adults	<48 km	40 days	2010–2012	↑ WBCs, PLTs, Hb, HCT, BUN, ALP, AST and ALT
D’andrea et al. [59]	October-17	Texas City, USA	791 smokers (733 exposed)	Adults	<48 km	40 days	2010–2012	↑WBCs, PLTs, ALP, AST and ALT
D’andrea et al. [60]	February-14	Texas City, USA	312 (157 exposed)	Children (<17)	<48 km	40 days	2010–2012	↓ WBCs; ↑ PLTs, ALP, AST, ALT and creatinine; Neurological, respiratory and dermatological symptoms
D’andrea et al. [61]	March-16	Texas City, USA	899 (641 exposed)	Children (<17)	<48 km	40 days	2010–2012	↓WBCs, HCT, Hb and BUN; ↑ PLTs, ALP, AST and ALT
Lee et al. [69]	November-02	Ulsan, Korea	192 (97 exposed)	Children (8–11)	<16 km	4–11 years	2000	In April measurement: ↓ WBCs, absolute neutrophil and lymphocyte count, RBCs, PTLs and Hb. In July measurement: no differences. In October measurement: ↓ RBCs and Hb.
Pelallo-Martinez et al. [70]	July-14	Coatzacoalcos, Mexico	102	Children (6–12)	~7 km	6–12 years	NS	Negative correlation between urinary t,t-MA and WBCs and RBCs count
Chen et al. [72]	April-19	Nanjing, China	421 (240 exposed)	Adults (50–71)	~3 km	5–71 years	2016	↓ Neutrophil counts, RBC counts, Hb concentration, HCT percentage, MCHC levels and PLT counts; ↑ monocyte and basophil counts
Li et al. [73]	June-19	Jilin, China	499	Adults	NS	>5 years	2016	↑ Detection rate of blood benzene; changes in urinary WBC related to benzene
Silva et al. [74]	August-19	Campos Elíseos district, Rio de Janeiro State, Brazil	190	Adults	<1 km	>3 months	2016–2017	Association between detectable S-PMA and variant allele in NQO1 with hematological abnormalities
*Epidemiological studies*								
Barregard et al. [83]	November-09	Lysekil, Sweden	NS	NS	<5 km	NS	1975–2004	↑ Incidence of leukemia
Axelsson et al. [84]	September-10	Stenungsund, Sweden	NS	NS	NS	NS	1974–2005	No evidence of increased risk of leukemia and lymphoma
Beale et al. [85]	September-10	Utah, USA	NS	NS	<5 km	NS	1973–2006	Increased risk of NHL; no evidence of increased risk of HL, MM and leukemia
Hurtig et al. [86]	2004	Ecuador	357,000 ca	Children (0–14)	NS	NS	1985–2000	↑ Risk of childhood leukemia
Weng et al. [87]	January-08	Taiwan	NS	Children (0–19)	NS	NS	1995–2005	↑ Incidence of childhood leukemia
Yu et al. [88]	August-06	Taiwan	NS	All (0–19; 20–29)	657.1 km^2^	NS	1997–2003	↑ Risk leukemia risk in 20–29 y.o. subjects
Lyons et al. [89]	April-95	Baglan Bay, South Wales	NS	0–24	1.5–3 km	NS	1974–1991	No augmented incidence of leukemias and lymphomas
Sans et al. [90]	April-95	Baglan Bay, South Wales	115,000 ca	All	7.5 km	NS	1974–1984 (incidence) 1981–1991 (mortality)	Augmented incidence of all tumors
Micheli et al. [91]	December-14	Falconara, Italy	526 (177 HM related deaths)	NS	65 km^2^	NS	1994–2003	↑ Risk of death from leukemia or NHL in females and retired-homemaker-unemployed category
Salerno et al. [92]	2013	Cerano, Italy	NS	NS	<3 km	NS	2003–2009	↑ Incidence of MM in women and leukemic lymphoid neoplasm in women and men
Dahlgren et al. [93]	November-08	Sugar Creek, Missouri	4500 ca	NS	1.6 km	NS	NS	↑Prevalence for Hodgkin’s disease
Ramis et al. [94]	February-12	10 site in Spain	1,744,988	NS	10 km	NS	1997–2006	Possible increased risk of NHL mortality, no gradient with increasing distance

**Table 2:** Main hypersensitivity studies and their characteristics found through our research.

Author	Date	Refineries Sites	Cohort	Age	Distance from Refineries	Exposure Time	Observational Time	Outcome in Exposed Groups
Smargiassi et al. [97]	July-14	Montreal, Quebec	72 recruited	Children (8–12)	NS	NS	10 days	Suggestion for a small ↓ in respiratory function with total concentrations of PAHs
Wichmann et al. [98]	March-09	La Plata, Argentina	1212 recruited	Children (6–12)	~10 km	NS	2005–2006	↑ Prevalence of asthma, ↑ asthma exacerbations, ↑ respiratory symptoms
De Moraes et al. [99]	August-10	Rio Grande do Norte, Brazil	209 recruited	Children (0–14)	within a 5-km radius	at least one year	1 year (2006)	↑ Frequency of respiratory symptoms
Rusconi et al. [100]	February-11	Sarroch, Sardinia	300 recruited	Children (6–14)	NS	NS	January–June 2007	↓ Lung function, ↑ markers of bronchial inflammation and oxidative DNA damage, ↑ MDA–dG adduct values
Idavain et al. [101]	February-19	Ida-Viru county, Estonia	1326 recruited	Children (8–12)	NS	NS	NS	↑ Prevalence of respiratory symptoms and of high fractional exhaled NO (FeNO) values (≥30 ppb)
Orru et al. [102]	February-18	Ida-Viru County, Estonia	5250 recruited	Adults (18–70)	NS	NS	2001–2013	↑ Frequency of wheezing, chest tightness, shortness of breath, asthma attack, long-term cough, cardiovascular diseases and diabetes in oil shale workers compared with exposed residents
Tanyanont et al. [105]	January-12	Map Ta Phut Municipality, Thailand	24.980 recruited	All (>13)	10 km radius	at least 1 year	September 2006–August 2007	↑ Frequency of all acute respiratory symptoms among residents living < 5 km, ↑ risk of dyspnea and wheezing in residents longer than 5 years near the industrial complex
Rovira et al. [106]	August-14	Tarragona county, Catalonia, Spain	5.196 recruited	Children (6–7; 13–14)	10 km	NS	2010	Not found higher prevalence of asthma, allergic symptoms and lower lung function. Significantly ↑ prevalence of respiratory hospitalizations and nocturnal cough

## Data Availability

Not applicable.
